# Mimicking Paracrine TGFβ1 Signals during Myofibroblast Differentiation in 3D Collagen Networks

**DOI:** 10.1038/s41598-017-05912-x

**Published:** 2017-07-18

**Authors:** Michael Ansorge, Jiranuwat Sapudom, Marina Chkolnikov, Martin Wilde, Ulf Anderegg, Stephanie Möller, Matthias Schnabelrauch, Tilo Pompe

**Affiliations:** 1 Universität Leipzig, Institute of Biochemistry, Johannisallee 21/23, 04103 Leipzig, Germany; 2 Universitätsklinikum Leipzig, Department of Dermatology, Venereology and Allergology, 04103 Leipzig, Germany; 3 Biomaterials Department, INNOVENT e. V., Prüssingstr. 27B, 07745 Jena, Germany

## Abstract

TGFβ1 is a key regulator for induction of tissue remodeling after dermal wounding. We present a model of paracrine delivery of TGFβ1 for differentiation of dermal fibroblasts based on a fibrillar 3D collagen matrix and embedded TGFβ1 releasing microparticles. We found differentiation into myofibroblasts was achieved in a TGFβ1 dependent manner at much lower doses than systemic delivery. This effect is accounted to the slow and sustained TGFβ1 release mimicking paracrine cell signals.

## Introduction

The understanding of wound healing on a cellular level is pivotal to prevent unwanted outcomes like increased scar formation or fibrosis during wound healing. After dermal wounding, regenerative processes start immediately to quickly close the wound and slowly restore tissue integrity. Wound healing is tightly regulated by different cell types, many cytokines, and involves interactions with the extracellular matrix (ECM)^1^. After initial wound closure by a fibrin clot, resident dermal fibroblasts and putative precursor cells are attracted towards the wounding site. These deposit ECM proteins and exert forces on the existing ECM leading to tissue contraction. Thereby the surrounding ECM becomes stiffer and the pre-stressed matrix lead to a transformation of fibroblasts into proto-myofibroblasts containing actin stress fibers^1^. The pre-stressed matrices demand stronger traction forces to ensure wound closure. For this reason, proto-myofibroblasts differentiate into myofibroblasts. These cells possess a pronounced cytoskeleton, an enhanced production of ECM molecules (e.g. collagen I and III, fibronectin and proteoglycans) and a strong capacity for tissue contraction, which is achieved by incorporation of alpha smooth muscle actin (αSMA) into their actin stress fibers^1^. αSMA incorporation is one of the most prominent markers of myofibroblast differentiation^2^.

TGFβ1 is known to be a key player in wound healing, particularly in myofibroblast differentiation^1, 3, 4^. This pro-inflammatory and heparin-binding cytokine is secreted by immune cells and proto-myofibroblasts as well as myofibroblasts in a temporarily defined, paracrine and autocrine manner^3, 5^. But lacking resolution of TGFβ1 release, sustained inflammation, and disturbed Smad (intracellular signal transducers of TGFβ1 signaling) signaling cause myofibroblasts to contract and produce ECM over prolonged time periods, resulting in hypertrophic scars, excess fibrous tissue, fibrosis and related loss of tissue function^1^. Hence, TGFβ1 is also used as a target for prevention of fibrotic diseases^6^.

Due to the high level of complexity *in vivo* and difficult access for high-resolution analytical tools, simplified *in vitro* models are necessary for in-depth understanding of TGFβ1 signaling and the development of therapeutic strategies. Such model systems have to closely mimic the *in vivo* situation to allow for physiologically relevant results and a reduction of ethically controversial animal studies. In the last decade it became evident that cell culture conditions in a 3D ECM context are needed, but are not provided by standard plastic dish cell culture^7^. Hence, such model systems should not only mimic the soft, fibrillar network characteristics of the ECM, but must also enable controlled delivery of mediators and cytokines.

**Figure 1:**
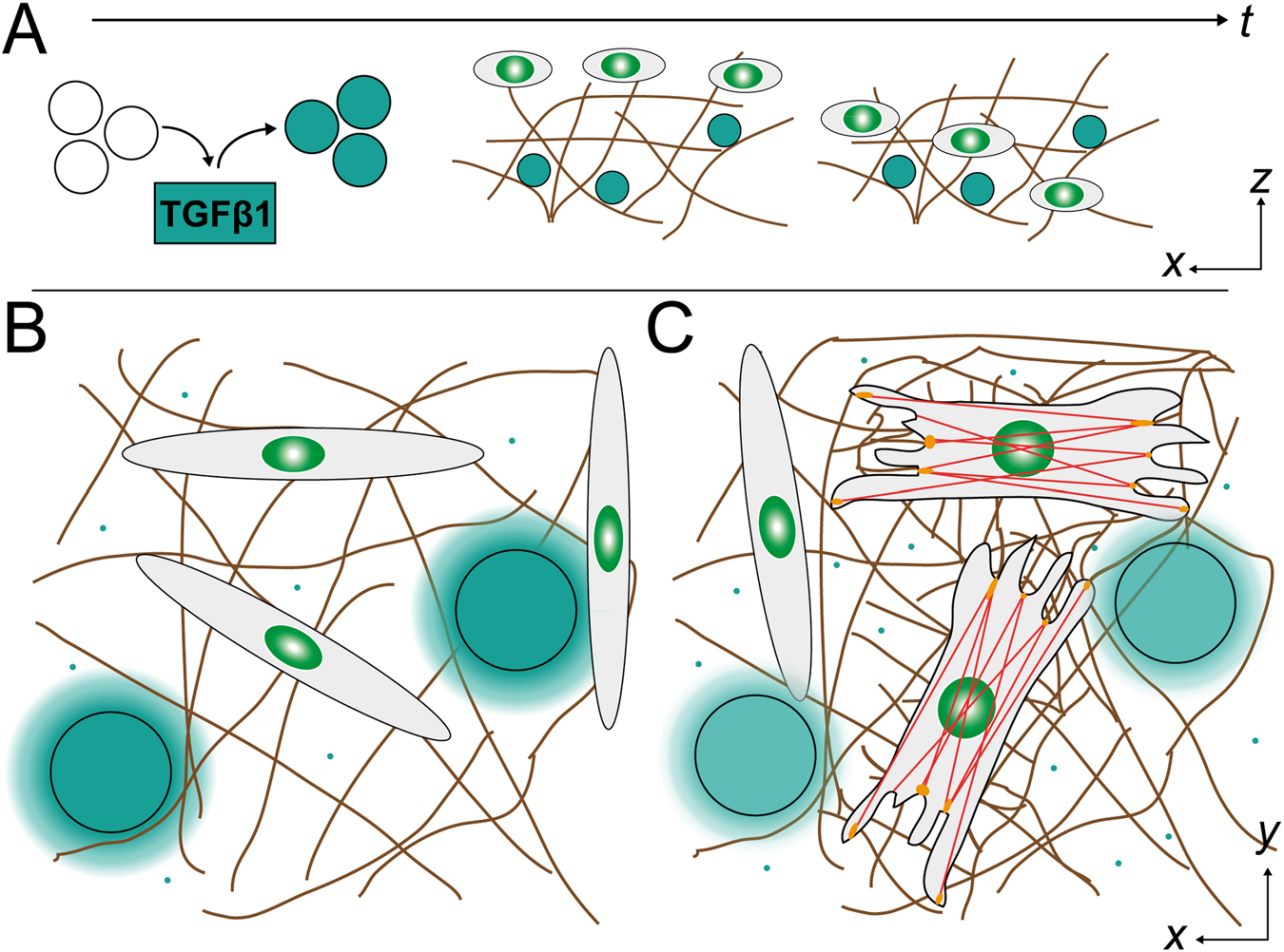
Scheme of experimental setup. (**A**) Time course of cell experiments. Prior to experiments GAG-functionalized µ-beads were laden in TGFβ1 (cyan) solutions with defined loading concentrations. TGFβ1-laden µ-beads were already present during collagen network (brown) reconstitution and immobilized in the fibrillary network. After overnight equilibration of the network with cell culture medium, fibroblasts were seeded on top of the collagen networks. This is the start of cell experiments. (**B**) In the beginning fibroblasts are more elongated and apparently have no αSMA incorporated into their cytoskeleton. The cells are embedded in a 3D collagen network (brown). (**C**) After stimulation by TGFβ1 (cyan small dots) delivered from µ-beads (cyan large dots) fibroblasts transform into tissue-producing and -contracting myofibroblasts increasing network’s stiffness. They have a prominent cytoskeleton with incorporated αSMA (red) to connect focal adhesions (orange), which transfer the forces to the extracellular matrix.

Here such a model system is introduced. It includes two main features of regenerating dermal tissue: i) the 3D soft, collagenous fibrillar ECM and ii) a paracrine release of TGFβ1. As depicted in Fig. 1A our model system is based on reconstituted 3D collagen I networks with a pore size of 5 to 10 micrometers^8^. These networks exhibit a fibrillar microstructure and a pore size similar to dermal ECM^9^. To deliver TGFβ1 in a localized, slow and sustained manner, a release system based on glycosaminoglycan (GAG) functionalized agarose microbeads (µ-beads) was used, as recently introduced for the chemokine SDF-1^10^. Differentiation of dermal fibroblasts into myofibroblasts within the 3D biomimetic matrices was determined by staining of TGFβ1 downstream target Smad2/3, which is phosphorylated and afterwards translocated to the nucleus, and αSMA incorporation into the actin cytoskeleton as well-known marker of contractile myofibroblasts (Fig. 1B and C)^2^.

## Results and Discussion

**Figure 2:**
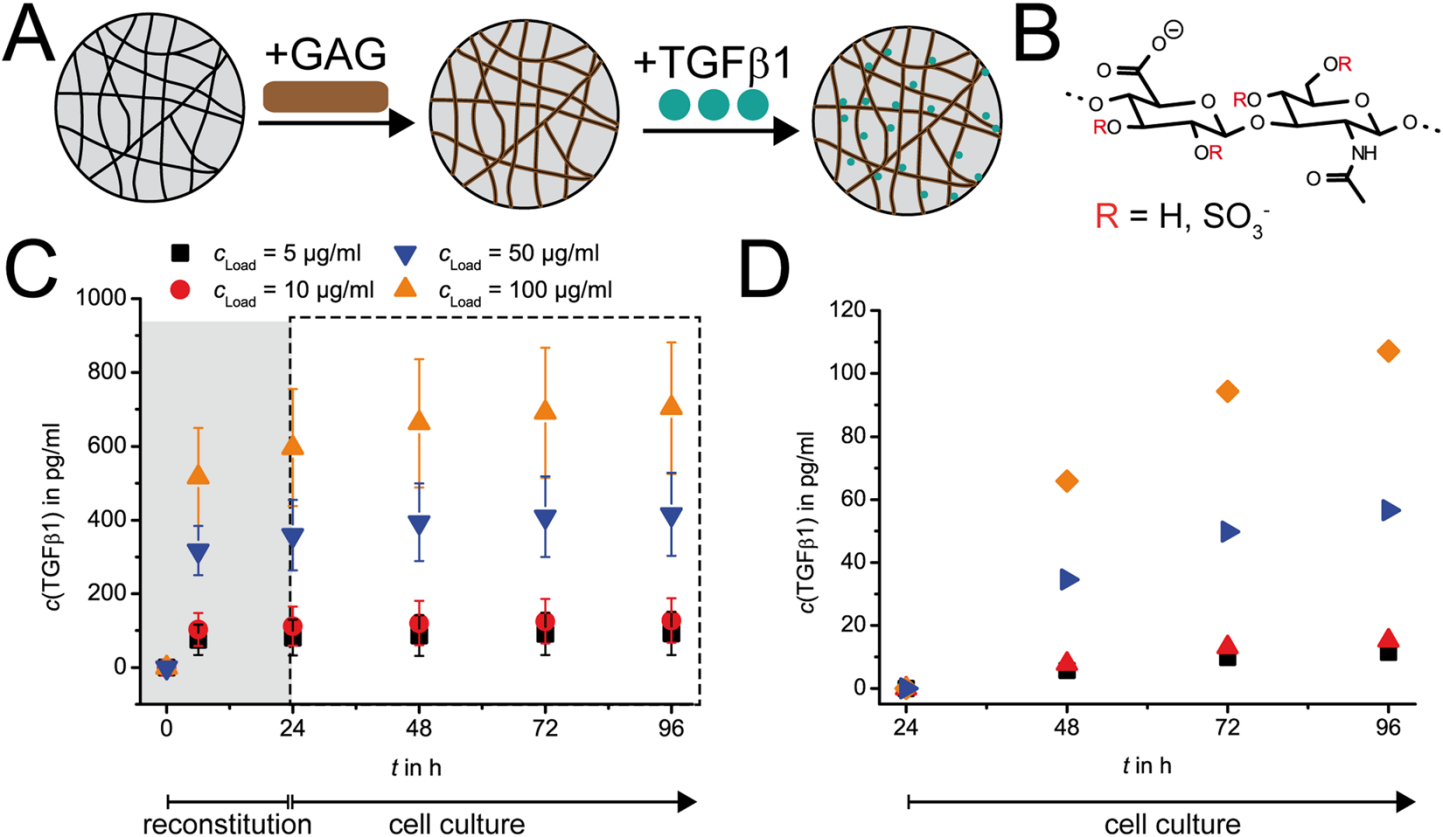
TGFβ1 is bound and released by µ-beads. (**A**) Modification of porous agarose µ-beads with GAG is achieved by reaction in presence of EDC, enabling formation of covalent bonds between GAG’s acidic groups and amine groups of µ-beads. The GAG provides binding sites for TGFβ1 adsorption. TGFβ1 adsorption is concentration dependent. Release is driven by a concentration difference between µ-beads’ interior and outside. (**B**) The GAG used in this study was medium-sulfated hyaluronan (msHA). The degree of sulfation is about 2 sulfate groups per disaccharide unit of the GAG and therefore similar to heparin. Possible positions of sulfate groups are marked with red ‘R’. (**C**) Release kinetics of TGFβ1 as determined by ELISA. Concentration of TGFβ1 was measured in the supernatant released from 10^4^ µ-beads. The depicted release curve is already re-calculated for cell culture conditions. It shows concentration increase of TGFβ1 in the supernatant delivered by 500 µ-beads. Released concentrations correlate with loading concentrations. The area shaded in grey (first 24 h) covers so-called “initial burst” of TGFβ1. This amount is washed out before cell culture experiments by repeated rinsing of collagen networks before seeding fibroblasts. (**D**) Increase of TGFβ1 concentration in the medium during cell experiments starting after cell seeding. The released concentration of TGFβ1 release over 24 h was subtracted. Release curves show TGFβ1 delivered by 500 µ-beads over 3 d from day 1 until day 4.

First we proved the TGFβ1 release characteristic of our established µ-bead system^10^. µ-beads were covalently functionalized with a chemically sulfated hyaluronan, thereby creating binding sites for proteins like TGFβ1 (Fig. 2A). This chemically sulfated hyaluronan with approximately two sulfate groups per disaccharide repeating unit (msHA) is similar to heparin concerning charge density and cytokine binding behavior (Fig. 2B). msHA showed high affinity for TGFβ1 in previous studies already^11^. As we already discussed in our last paper, available binding sites introduced by GAG functionalization inside the µ-beads exceed binding sites occupied by TGFβ1 even at high cytokine concentrations^10^. µ-beads were laden overnight in TGFβ1 solutions with concentrations of 5, 10, 50 and 100 µg/ml. TGFβ1 release from 10^4^ µ-beads into 180 µl of 1 wt-% bovine serum albumin (BSA) in PBS was determined over 4 days by ELISA. BSA was inserted to mimic culture conditions like serum presence. The (already re-scaled, see below) results (Fig. 2C) show that variation of loading concentrations permits control of concentrations of bound cytokine in the µ-beads and consequently the release of TGFβ1 into the medium. Such a control of TGFβ1 release was also found in another heparin-based layered hydrogel system^12^.

Importantly, TGFβ1 was released in a sustained manner over several days from the µ-beads besides high release rates during the first 24 h. This phase is usually called ‘initial burst’. This behavior is characteristic for affinity-based release systems and can be fitted with simplified diffusion models^10, 13, 14^. Thus, TGFβ1 mobility inside the µ-beads can be estimated with a diffusion coefficient of 5 ∙ 10^−4^ µm^2^/s. This value is comparable to diffusion coefficients of chemokines in alginate µ-beads^14^. It indicates a tremendous reduction of TGFβ1 mobility compared to free diffusion in solution (usually about 10^2^ µm^2^/s for small cytokines)^15^. Due to the high affinity of TGFβ1 to the GAG inside the µ-beads, a continuous binding and re-binding of TGFβ1 and msHA leads to a reduced effective diffusion. As the diffusion coefficient does not depend on loading concentration, protein-protein interaction inside the µ-particles, especially pore blocking effects, can be excluded^16^. On the contrary, this suggests the existence of free binding sites, which are able to store and release TGFβ1 even at higher concentrations. In sum, we are able to deliver low amounts of TGFβ1 in the concentration range of 10 to 100 pg/ml over a period of several days in a sustained manner into 3D cell culture environment. Such low and sustained release rates nicely mimic paracrine cell signals of cytokine release in the range of 10^−7^ ng/h^14, 17^.

Next, we set off to prove the efficacy of our TGFβ1 delivery system for fibroblast differentiation into myofibroblasts. We incorporated differentially laden µ-beads into 3D fibrillar collagen I networks and cultivated human foreskin-derived dermal fibroblasts on these matrices (Fig. 1A). Such 3D collagen I networks with pore sizes in the range of 5 to 10 µm were already shown to enable good migration of fibroblasts into the 3D matrix and successful cell culture over several days^18, 19^. The released concentrations in Fig. 2C were recalculated out of the measured release data to be comparable to cell culture conditions with 500 µ-beads incorporated in the matrices and supplemented with 500 µl medium. This presentation indicates available TGFβ1 at concentration levels of some hundreds pg/ml in our system. However, importantly the resulting TGFβ1 concentration available in cell experiments was much lower. This is because TGFβ1-laden µ-beads are added already during collagen reconstitution (Fig. 1A), and are present during medium conditioning and exchange (gray area in Fig. 2C) 24 h before the start of cell culture (white area in Fig. 2C). The washing steps during this preparation period removed the TGFβ1 released during the ‘initial burst’ and thus reduced TGFβ1 concentration available during cell culture. Hence, cumulative amounts of TGFβ1 released by µ-beads during the cell culture can be stated as 10, 15, 55, 110 pg/ml for loading concentrations of 5, 10, 50, 100 µg/ml, respectively (Fig. 2D). This linear dependency also underlines excess of available GAG binding sites compared to bound TGFβ1 molecules even at highest loading concentration used.

**Figure 3:**
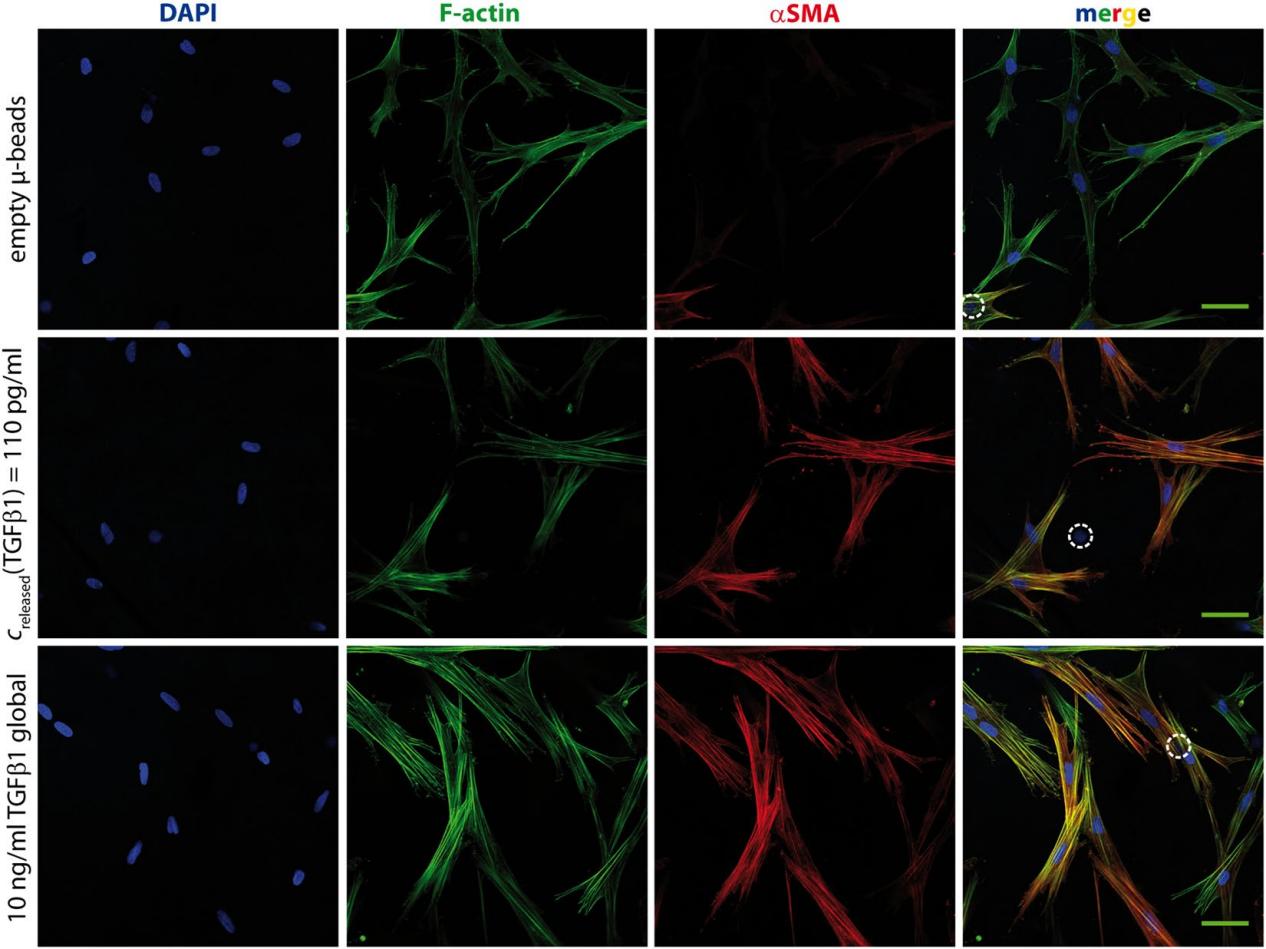
αSMA is expressed in actin stress fibers after stimulation with TGFβ1. Non-TGFβ1 stimulated fibroblasts (“empty beads”, upper row) exhibit phalloidin-stained stress fibers (green), but negligible staining of fibrillary αSMA (red). In comparison, TGFβ1 stimulated cells show αSMA staining in F-actin stress fibers. Colocalization of F-actin and αSMA is obvious in the merged images by yellow fibrillar structures. TGFβ1 delivery – whether 10 ng/ml systemic (lower row) or sustained local release of 110 pg/ml from µ-beads (middle row) – leads to comparable results. Scale bar: 50 µm. White dashed circle in the merged images indicates µ-bead position.

In the presence of bioactive TGFβ1, fibroblasts differentiate into myofibroblasts. This differentiation is accompanied by a change in morphology from more elongated shape towards a more widespread shape along with the formation of pronounced actin stress fibers. As a prominent and widely accepted marker of myofibroblast differentiation, αSMA was stained since it is highly expressed after some days of myofibroblast differentiation^20^. In initial experiments, we proved incorporation of αSMA in F-actin stress fibers after TGFβ1 stimulation by anti-αSMA antibody and phalloidin (Fig. 3, red and green, lower row). Similarly, µ-bead-delivered TGFβ1 was able to induce differentiation, but at much lower concentrations (Fig. 3, red and green, middle row). In absence of TGFβ1, F-actin and some stress fibers were also visible but αSMA was not present in actin stress fibers, except for very few cells (Fig. 3, upper row). In further experiments, we only used αSMA staining in stress fibers as marker of myofibroblast differentiation.

**Figure 4:**
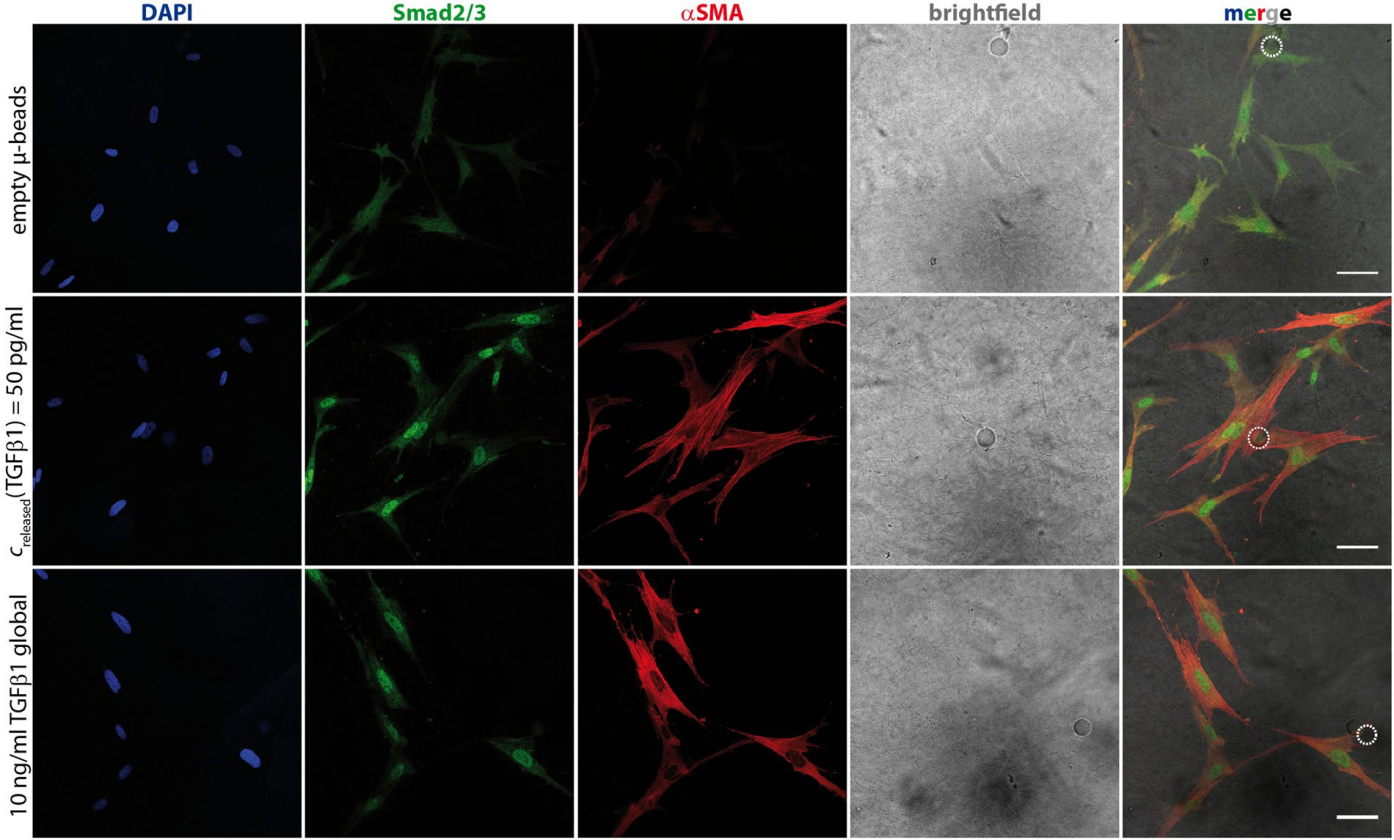
Nuclear Smad2/3 is expressed after stimulation with TGFβ1. Non-TGFβ1 stimulated fibroblasts (“empty beads”, upper row) exhibit only similar faint Smad2/3 staining in nucleus and cytosol. In comparison, TGFβ1 stimulated cells show strong nuclear Smad2/3. Correspondingly, actin stress fibers show αSMA staining in case of TGFβ1 stimulation. TGFβ1 delivery – whether 10 ng/ml systemic (lower row) or sustained local release of 55 pg/ml from µ-beads (middle row) – leads to comparable results. Scale bar: 50 µm. White dashed circle in the merged images indicates µ-bead position.

Smad2/3 phosphorylation and nucleic translocation is well-known as another downstream target of TGFβ receptor type I and type II activation. After TGFβ1 binding to its receptors, Smad2/3 get phosphorylated, bind to Smad4 and the complex translocates from the cytoplasm into the nucleus^21^. Immunostaining of Smad2/3 allows the identification of cells with activated TGFβ1 signaling by accumulation of phosphorylated Smad2/3 inside the nucleus (Fig. 4, green).

Figure 4 (lower row) shows typical myofibroblasts, displaying the widespread morphology, αSMA incorporation into actin stress fibers and Smad2/3 staining inside the nucleus. In contrast, fibroblasts not stimulated with TGFβ1 (Fig. 4, upper row) exhibited a slightly elongated morphology without prominent αSMA^+^ fibers. Smad2/3 staining intensity was similar in cytosol and nucleus suggesting weak Smad-mediated gene expression in these cells. Within the samples of µ-bead-delivered TGFβ1, cells looked very similar to the positive control regarding αSMA incorporation and Smad2/3 staining (Fig. 4 middle row). This behavior was observed at much lower concentrations indicating an advantage of paracrine delivery.

**Figure 5:**
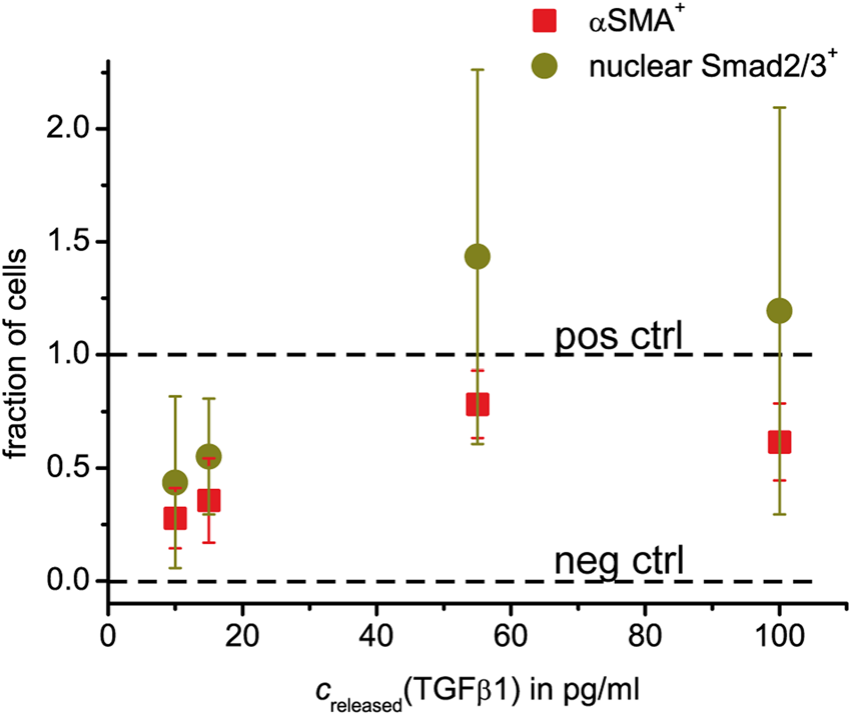
TGFβ1 delivery in paracrine manner leads to dose-dependent myofibroblast differentiation. Myofibroblast differentiation correlates with TGFβ1 loading concentration of µ-beads and thereby with delivered TGFβ1 concentration. Myofibroblast differentiation was quantified by Smad2/3 nucleus (nuclear Smad2/3^+^) and fibrillar αSMA (αSMA^+^) staining. Fraction of Smad2/3^+^ and αSMA^+^ cells were normalized to *positive control* (10 ng/ml TGFβ1 systemic) as 1 and *negative control* (no additional TGFβ1) as 0. Data are presented as mean ± standard deviation. Experiments were done in triplicate, each experiment with fibroblasts from a different donor. For each condition, about 100 cells were investigated.

For quantification of myofibroblast differentiation, we counted cells positive for fibrillary arranged αSMA (termed αSMA^+^) and nuclear Smad2/3 (termed nuclear Smad2/3^+^) for every condition (TGFβ1 loading concentration of µ-beads (5, 10, 50, 100 µg/ml, positive and negative control)). Primary fibroblasts are known to be intrinsically heterogeneous with subpopulations of higher fibrogenic potential^22^, and to have a strong donor variability. Therefore, we normalized the ratio of positive-counted cells to total cells such that the ratio of the positive control (empty µ-beads and 10 ng/ml TGFβ1 systemically given in cell culture medium) was set to 1 and the ratio of the negative control (empty µ-beads and cell culture media without TGFβ1 added) was set to 0. We found an increasing fraction of differentiated cells (for both markers, αSMA^+^ and nuclear Smad2/3^+^) with increasing delivery concentration of TGFβ1 (Fig. 5). At high loading concentrations (50 and 100 µg/ml) corresponding to concentrations of cumulatively released TGFβ1 of 55 and 110 pg/ml, respectively, cell differentiation saturated at a level comparable to the positive control. Most importantly, the concentrations of cumulatively released TGFβ1 from the µ-beads (10 to 100 pg/ml) were 2 to 3 orders of magnitude smaller than the 10 ng/ml (10^4^ pg/ml) in the positive control of systemic supplement in the cell culture medium, compare also Figs. 3 and 4, middle and lower row.

How can these low concentrations of TGFβ1 in the pg/ml range induce similar myofibroblast differentiation as a systemic 10 ng/ml stimulation? Comparable dose-dependent experiments at standard cell culture conditions did not indicate relevant myofibroblast differentiation at such low concentrations of TGFβ1^23^. We attribute this effect to the two important functional properties of our µ-bead release system: i) the sustained release of TGFβ1 over several days in combination with ii) protection of TGFβ1 from proteolytic degradation due to GAG binding inside the µ-beads^10, 24^. The first feature imitates the continuous production of TGFβ1 by neighboring cells, while the second feature mimics functions of *in vivo* ECM^25^. Both functional properties together permit the delivery of small amounts of TGFβ1 in its biologically active form, persistent over time periods of several days, exactly mimicking the *in vivo* situation of constant paracrine TGFβ1 secretion by neighboring cells^5^.

In support of our arguments on the mode of action, fibroblast differentiation at low TGFβ1 levels was already discussed in the literature for another GAG-containing bulk hydrogel system, however, without showing supporting data^12^. Furthermore, we want to discuss an additional option for the operational mode of our system. A report in the literature indicates that only a short contact time of 30 min between TGFβ1 and fibroblasts is needed for sustained up-regulation of genes involved in fibrosis^26^. In this way, high initial TGFβ1 concentration induces cell differentiation and propagation of the stimulus by autocrine TGFβ1 production^23^. Hence, the sustained delivery of TGFβ1 in our system could also persistently re-stimulate autocrine TGFβ1 production and thus lead to an amplification of the stimulus of our low TGFβ1 concentrations. However, we could show in our lab that autocrine TGFβ1 stimulation is insufficient for maintaining differentiation state in the fibrillar 3D collagen matrix as removal of TGFβ1 from myofibroblasts’ growth medium led to the disappearance of αSMA^+^ cells^27^. Therefore, we attribute the observed differentiation to the constant delivery of TGFβ1 from µ-beads.

Compared to our previous work^10^, where short-range cytokine gradients were formed with these µ-beads, we did not observe any spatially constrained differentiation in the vicinity of the µ-beads. In all conditions, cell differentiation was independent of distance to TGFβ1-delivering µ-beads. We explain this by the fast TGFβ1 diffusion from the µ-beads in the cell culture medium (time scale of minutes) in comparison to the slow cell differentiation (time scale of days). This difference in time scales leads to a ‘fast’ adjustment of a low uniform background concentration of delivered TGFβ1 in medium between µ-beads (10 to 100 pg/ml), followed by the ‘slow’ differentiation of myofibroblasts with αSMA expression. Only very adjacent to the µ-beads (roughly 20 µm) higher concentrations are expected in a gradient manner^10^.

## Conclusion

In sum, our setup based on cytokine delivery by µ-beads simulates paracrine signaling in a 3D biomimetic environment. Cytokine delivery during wound healing *in vivo* is achieved by different cell types like macrophages, platelets and fibroblasts which constantly secrete cytokines into the surrounding tissue^5^. Cytokine proteolysis is a minor issue due to constant production and secretion by source cells. In contrast to usual cell culture, where cytokines are given globally and mostly at once, we present a biomimetic approach containing fibrillar ECM and surrogates for cytokine secreting cells with defined release characteristic over several days.

We focused on TGFβ1 delivery because it is the key cytokine for transformation of tissue-resident fibroblasts into tissue-contracting and -remodeling myofibroblasts, which enable wound closure with therapeutic relevance. Within the presented model system, we are able to mimic early stages of wound healing. The mode of action of our setup leads to a concentration-dependent fibroblast differentiation into myofibroblasts at very low TGFβ1 concentration levels. In addition, the system might be expanded towards other cytokines also investigating putative interactions between functionally competing pro-inflammatory and anti-inflammatory cytokines. Delivery is possible by vehicles of different size to distinguish release of two different cytokines. A combination of local release and global constant cytokine background can be implemented, too. The latter can be introduced sequentially, to simulate different time phases of wound healing. IL10 might be a relevant candidate for manipulating TGFβ1 induced effects at late stages of wound healing, like overshooting scarring^28, 29^.

## Methods

### GAG synthesis

The medium-sulfated hyaluronan (msHA) was synthesized by transforming hyaluronan (sodium salt, Aqua Biochem, Dessau, Germany) into its tetrabutylammonium salt and sulfating the latter with SO_3_/pyridine as already described in detail^30^. The estimated degree of sulfation (average number of sulfate groups per disaccharide repeating unit) of msHA was 2.3 and the weight average molecular weight determined by gel permeation chromatography with laser light scattering detection mode was 23.9 kDa.

### µ-bead functionalization

Functionalization of crosslinked 4% agarose microbeads (µ-beads, mean diameter 17 µm; ABT, Madrid, Spain) with glycosaminoglycans (GAG) was achieved as previously described^10^. Briefly, 200 µl of µ-bead suspension was washed thrice with water alternating with centrifugation (1000 × *g*, 5 min). Washed µ-beads were mixed with 0.1 M N-(3-dimethylaminopropyl)-N′-ethylcarbodiimide (EDC; Merck, Darmstadt, Germany) and msHA and reacted at room temperature for 4 h. Afterwards µ-beads were washed with water, 1 M sodium chloride (NaCl; Applichem, Darmstadt, Germany) and again with water. The resulting suspension was stored in phosphate buffered saline (PBS; Biochrom, Berlin, Germany) with 0.02% sodium azide (NaN_3_; Applichem) at 4 °C.

### TGFβ1 release kinetics

The release profile of TGFβ1 (Peprotech, Hamburg, Germany) was determined using the supernatant of µ-bead suspensions. About 10^4^ µ-beads were incubated in TGFβ1 solution in PBS with a concentration of 100, 50, 10, 5 µg/ml at 4 °C overnight. The µ-beads were washed thrice with PBS supplemented with 1 wt-% bovine serum albumin (BSA, Sigma-Aldrich) to imitate cell culture conditions (alternating centrifugation and re-suspending in PBS +1 wt-% BSA, supernatant was discarded). Cytokine-laden µ-beads were stored in 180 µl PBS +1 wt-% BSA at 37 °C. After spinning down the µ-bead suspension (1000 × g, 5 min) 150 µl of supernatant was collected and replaced by fresh solution. Supernatants were frozen at −20 °C until all samples were collected. In order to achieve kinetics of release, supernatants were harvested initially after washing and after 6, 24, 48, 72, 96 h. For control reasons a TGFβ1 standard solution was also similarly treated and investigated to determine TGFβ1 degradation over time, indicating no significant degradation in the experimental setup.

TGFβ1 concentrations of the supernatants were determined by enzyme-linked immunosorbent assay (ELISA) for TGFβ1 (Affymetrix eBiosience, San Diego, CA, USA) according to the manufacturer’s instructions. Absorbance at 450 nm was quantified by Infinite F200 PRO plate reader (Tecan, Männedorf, Switzerland). Samples were analyzed in triplicate. Results were averaged and presented as mean ± standard deviation.

TGFβ1 mobility inside the µ-beads was described by a diffusion model. Based on Fick’s 2^nd^ law for spherical devices the diffusion coefficient of TGFβ1 inside the µ-beads was calculated. Fitting release curves with the following equation allowed determination of *D*_Bead_ as described previously^10^.

$$\frac{c}{{c}_{\infty }}=1-\frac{6}{{{\rm{\pi }}}^{2}}\sum _{n=1}^{\infty }\frac{1}{{n}^{2}}\exp (-\frac{{D}_{{\rm{Bead}}}{n}^{2}{{\rm{\pi }}}^{2}t}{{a}^{2}})$$

Equation was solved for *n* ≤ 7. Radius of the µ-beads is given by *a*, *t* is particular time point and *c* and *c*_∞_ are concentrations at particular time point *t* and asymptotic achieved release concentration after infinite amount of time, respectively.

### Reconstitution of collagen I matrices

Collagen I matrices with a pore size ranging between 5 and 10 µm were reconstituted and characterized with already published protocols^8, 19, 31^. Briefly, 13 mm coverslips (VWR International, Darmstadt, Germany) were cleaned, functionalized with 3-aminopropyltriethoxysilane (Alfa Aesar, Karlsruhe, Germany) to enable subsequent covalent binding of poly(styrene-*alt*-maleic anhydride) (PSMA; MW 30 000 g/mol, Sigma-Aldrich, Steinheim, Germany) monolayers. On top 3D collagen I matrices were reconstituted as previously described from rat tail collagen solutions (stock concentration 4.1 mg/ml, lot# 3298599, Corning, Amsterdam, Netherlands)^8^. Fibrillogenesis took place in phosphate buffer at pH 7.5 with a collagen concentration of 2 mg/ml at 37 °C for 1 h. Collagen matrices were washed three times with PBS and equilibrated with cell culture medium overnight prior to cell seeding.

### Cell culture

Dermal fibroblasts from human foreskin were harvested as previously described after informed consent^32^. Cells were expanded on usual tissue culture plastic (Greiner Bio-One, Frickenhausen, Germany) and used for analysis until 4^th^ passage. Fibroblasts were cultured in Dulbecco’s modified Eagle’s medium (DMEM; Biochrom) supplemented with 10 vol% fetal bovine serum (Biochrom) and 1 vol-% Zellshield (antibiotic; Biochrom) at 37 °C in 5% CO_2_ in air at 95% humidity. For analysis of cell fates in 3D, equilibrated collagen matrices were place in 24 well plates (Greiner Bio-One) and 10^4^ fibroblasts were seeded on top of the matrices 24 h after reconstitution as described previously^18^.

### Immunocytostaining and imaging

In order to reveal differentiation, cells were stained in the collagen networks after 6 d. Cells were fixed with 4% paraformaldehyde (Carl Roth, Karlsruhe, Germany) and permeabilized with 0.1% Triton X-100 (Carl Roth). Cell nuclei were stained with DAPI (Invitrogen, Karlsruhe, Germany). Cytoskeletal actin was stained with Alexa 488-Phalloidin (Invitrogen) to investigate cell morphology and to prove co-alignment of F-actin and αSMA. Staining of Smad2/3 was done with a primary rabbit antibody (Cell Signaling Technology, Leiden, Netherlands) and subsequently donkey anti-rabbit IgG-CFL 488 antibody (0.4 mg/ml, Santa Cruz Biotechnology, Heidelberg, Germany). Staining of αSMA was achieved with efluor660 conjugated antibody (0.2 mg/ml, ebioscience, Frankfurt, Germany). Antibody staining was performed according to the manufacturer’s instructions.

For imaging coverslips were turned over and cell-laden networks placed face down in a 24-well plate with glass bottom (#1.5, deviation ± 5 µm, Greiner Bio-One) on a confocal laser scanning microscope (cLSM; LSM 700, Carl Zeiss, Jena, Germany) with 20×/0.8 Plan-Apochromat objective (Carl Zeiss). Imaging was performed deep in the collagen network at the level of the µ-beads. Cells remaining on the collagen matrix surface were excluded from imaging and subsequent analysis.

### Experimental setup of µ-bead-based TGFβ1 release in 3D collagen matrices

Collagen I matrices were reconstituted 24 h before cell seeding as described above with addition of 500 TGFβ1 laden µ-beads (related to one collagen matrix) into reconstitution solution. Positive control was global stimulation with TGFβ1 (10 ng/ml) whereas negative control was no stimulation with TGFβ1. For positive and negative control, collagen matrices were prepared with empty µ-beads. Prior to cell seeding, the medium was exchanged to deplete global concentration of already released TGFβ1. Afterwards cells were cultured for 6 d without passaging or medium exchange.

Images were analyzed manually. About 100 cells per condition were investigated. Total number of cells was counted using DAPI signal. Signal transduction of TGFβ1 in fibroblasts was investigated with Smad2/3. Positive cells showed brighter staining in the nucleus than in cytoplasm. Cells were classified as myofibroblasts only if they incorporated αSMA into their fibrillary cytoskeleton. Experiments were done in triplicate and results are presented as mean ± standard deviation.

### Data availability

The datasets generated and analyzed during the current study are available from the corresponding author upon reasonable request.
